# Phytochemical investigation and assessment of the anti-inflammatory activity of four *Heracleum* taxa growing in Turkey

**DOI:** 10.3389/fphar.2024.1494786

**Published:** 2025-01-06

**Authors:** Ekin Kurtul, Esra Küpeli Akkol, Büşra Karpuz Ağören, Büşra Yaylacı, Özlem Bahadır Acıkara, Eduardo Sobarzo-Sánchez

**Affiliations:** ^1^ Department of Pharmacognosy, Faculty of Pharmacy, Zonguldak Bülent Ecevit University, Zonguldak, Türkiye; ^2^ Department of Pharmacognosy, Faculty of Pharmacy, Gazi University, Ankara, Türkiye; ^3^ Department of Pharmacognosy, Faculty of Pharmacy, Başkent University, Ankara, Türkiye; ^4^ Department of Pharmacognosy, Faculty of Pharmacy, Ankara University, Ankara, Türkiye; ^5^ Centro de Investigación en Ingeniería de Materiales, Facultad de Medicina y Ciencias de la Salud, Universidad Central de Chile, Santiago, Chile; ^6^ Department of Organic Chemistry, Faculty of Pharmacy, University of Santiago de Compostela, Santiago de Compostela, Spain

**Keywords:** anti-inflammatory activity, apiaceae, carrageenan, coumarin, *Heracleum*

## Abstract

**Introduction:**

*Heracleum* L. has been known as “hogweed” and used for inflammatory diseases, including fever, enteritis, and bronchitis, for many years worldwide. The *Heracleum* genus is also prominently recognized for its high content of coumarins, which are considered a significant group of natural compounds known for their noteworthy anti-inflammatory properties.

**Methods:**

The present study evaluated the anti-inflammatory activity of dichloromethane and methanolic extracts from *H. paphlagonicum*, *H. sphondylium* subsp. *ternatum*, *H. sphondylium* subsp. *elegans*, and *H. sphondylium* subsp. *cyclocarpum* (100 mg/kg), which have not been previously investigated for their anti-inflammatory properties. Inflammation models induced by carrageenan, prostaglandin E2, and serotonin were employed to evaluate anti-inflammatory activity, using indomethacin (10 mg/kg) as the reference standard. Statistical differences between treatment and control groups were evaluated using ANOVA with Student-Newman-Keuls *post-hoc* tests. Additionally, the coumarin contents of the extracts were quantified as mg/g by high-performance liquid chromatography.

**Results and discussion:**

*H. sphondylium* subsp. *cyclocarpum* roots displayed the highest inhibition for carrageenan, prostaglandin E2, and serotonin-induced hind paw edema, with inhibition ranges of 22.8%–36.9%, 5.4%–35.7%, and 3.9%–17.9%, respectively, while the inhibition ranges for indomethacin were 12.8%–44.3%, 2.7%–41.3%, and 7.1%–30.6%, respectively. The highest bergapten and imperatorin quantities were found in *H. sphondylium* subsp. *cyclocarpum* roots (0.49% and 0.14%) and in *H. sphondylium* subsp. elegans roots, which had the highest xanthotoxin level (0.06%). Angelicin was detected in *H. paphlagonicum*, *H. sphondylium* subsp. *elegans*, and *H. sphondylium* subsp. *cyclocarpum* roots at concentrations of 0.04%, 0.04%, and 0.02%, respectively. The correlation between the highest inhibitory activity observed in *H. sphondylium* subsp. *cyclocarpum* roots and the elevated levels of coumarins, particularly bergapten and imperatorin, suggests a potential link between coumarin concentration and anti-inflammatory effects. Additionally, our findings support the traditional use of this genus for treating inflammatory disorders. Further investigations are necessary to identify the active compounds and elucidate the mechanisms of action of these plants, potentially leading to the discovery of novel therapeutic options for the treatment of inflammation.

## 1 Introduction

Inflammation is a defense mechanism developed to maintain the organism and stimulated by conditions such as infection and tissue damage. It causes reactions such as redness, swelling, pain, muscle weakness, and heat (Kara and Müdüroğlu, 2008; Medzhitov, 2008). Although the inflammatory response is usually beneficial for its protective activity against infection, irregular inflammation can lead to septic shock (Medzhitov, 2008), neurodegenerative diseases such as Alzheimer’s disease, cancer (Kara and Müdüroğlu, 2008), arterial hypertension, cardiovascular diseases, arthritis, osteoarthritis, asthma, gingivitis, diabetes, obesity, metabolic syndrome (Bansal et al., 2013; Marques-Rocha et al., 2015) and depression (Dantzer et al., 2008). These ailments reduce quality of life; therefore, decelerating the inflammatory process is significantly important, and non-steroidal anti-inflammatory drugs (NSAIDs) are commonly used to manage inflammation. However, NSAIDs have significant adverse effects such as gastrointestinal disorders, water retention, kidney failure, bronchospasm, and hypersensitivity. These harmful effects are particularly prominent during long-term treatment, which limits the therapeutic use of NSAIDs in treating chronic inflammation (Vonkeman and Van De Laar, 2010). The development of new drugs with minimal or no side effects is crucial. Medicinal plants have served as sources of medicine since ancient times to treat various diseases. Even today, natural products, particularly medicinal plants, continue to be an essential source for creating new drugs, drug leads, and chemical entities (Heinrich et al., 2012; Shen, 2015).

The *Heracleum* L. genus has been known as “hogweed” and is represented by more than 120 species around the world (Bahadori et al., 2016). *Heracleum* species have been used as spices and medicinal plants for many years, especially for treating inflammation and related diseases. Fruits and flowers of *H. persicum* Desf. ex Fisch., C. A. Mey. & Avé-Lall. are prepared as infusions and decoctions for migraine and sinus headache treatment in Iranian folk medicine (Amiri and Joharchi, 2016; Hosseinzadeh et al., 2019). *H. candicans* Wall. ex DC. and *H. yungningense* Hand.-Mazz. roots are used to reduce fever and *H. rapula* Franch. is used to relieve pain in Traditional Chinese Medicine (TCM). *H. rigens* Wall. ex DC. is another species used to treat skin problems and pain (Hosseinzadeh et al., 2019). *H. candicans* roots are employed as a tonic for eczema and itching in India (Rastogi et al., 2007). In south-eastern Serbia, the roots and leaves of *H. sphondylium* L. are recorded as useful agents in rheumatoid arthritis when applied externally (Jarić et al., 2015), and the roots are used against toothache by chewing in Italy (Vitalini et al., 2015). *H. humile* Sm. roots are prepared as paste and used for snakebites, fever, and abdominal cramps caused by worms in Lebanon (Arnold et al., 2015). Roots of *H. moellendorffii* Hance and *H. lehmannianum* Bunge are used for arthritis, backache, fever, and pain in Korea (Kim et al., 2019) and Afghanistan (Karimi and Ito, 2012).

In Turkish folk medicine, the utilization of members of the genus *Heracleum* has been recorded for various inflammatory diseases. *H. platytaenium* Boiss. leaves are used as aromatic baths for sunstroke; leaves and fruits are used against gastritis and enteritis (Cansaran and Kaya, 2010; Dincel et al., 2013). Decoction prepared from *H. trachyloma* Fisch. & C.A.Mey. stems is consumed to relieve stomachaches (Altundag and Ozturk, 2011). Leaves of *H. pastinacifolium* C. Koch are applied to the affected area for rheumatism (Güneş and Özhatay, 2011). Additionally, the leaves of *H. pastinacifolium* and *H. trachyloma* are used to treat asthma and bronchitis (Polat et al., 2015; Polat, 2019). *H. antasiaticum* Manden. leaves are used as a compress for wound healing (Tetik et al., 2013). Flowers of *H. humile* are used against headaches (Fakir et al., 2016).

Biological activity studies have demonstrated the antimicrobial, antiproliferative (Bahadori et al., 2016), antioxidant, and anti-inflammatory effects (Hajhashemi et al., 2009). Additionally, anti-Alzheimer’s, anti-neurodegenerative, antidiabetic, and antiviral activities have also been reported (Bahadori et al., 2016).

Phytochemical investigations have revealed the presence of various types of secondary metabolites in the *Heracleum* genus. Flavonoids such as astragalin, hyperoside, rutoside, kaempferol 3-*O*-rutinoside (nicotiflorin), isorhamnetin-3-*O*-rutinoside (narcissoside), and isorhamnetin-3-*O*-*β*-glucopyranoside have been identified in this genus (Bahadori et al., 2016; Gürbüz, 2019). Additionally, studies have shown that *Heracleum* species are rich in coumarin compounds including simple coumarins like scopoletin, umbelliferone, limettin, geijerin, dehydrogeijerin, suberosin, osthol, isophellodenol C, and yunngnin A-B (Nakamori et al., 2008; Bahadori et al., 2016). Furanocoumarins such as bergapten, isobergapten, allobergapten, psoralen, isopsoralen, imperatorin, isoimperatorin, alloimperatorin, alloisoimperatorin, pimpinellin, isopimpinellin, heraclenol, heraclenin, isoheraclenin, apterin, byakangelicin, byakangelicol, marmesin angelicin, sphondin, xanthotoxin, columbianadin, columbianetin and their derivatives, have also been isolated (Rastogi et al., 2007; Nakamori et al., 2008; Bahadori et al., 2016). A pyranocoumarin derivative 5,6-dihydropyrano-benzopyrone (Zhang et al., 2020) has also been identified in *Heracleum* species. Furthermore, lignans, alkaloids, lipids, and essential oils have been reported in the *Heracleum* genus (Bahadori et al., 2016; Zhang et al., 2017).

Coumarins form a fundamental structural framework found in many natural products and are widely recognized as a key core structure in medicinal chemistry. Numerous coumarin derivatives display notable biological activities, such as anticoagulant, antioxidant, antiangiogenic, anticancer, and antibacterial effects. The potential of the coumarin nucleus in the development of anti-inflammatory drugs has been thoroughly investigated in the literature, with numerous studies highlighting the anti-inflammatory effects of specific coumarin derivatives through various mechanisms (Rostom et al., 2022). In addition, flavonoids, which are among the most commonly encountered polyphenols in the plant kingdom, have been shown to possess antioxidant, cardioprotective, hepatoprotective, anti-inflammatory, anticancer, and antimicrobial effects (Kumar and Pandey, 2013). Many flavonoids help reduce inflammation and oxidative stress by influencing key cellular pathways, lowering inflammatory molecule levels, and reducing the activity of pro-inflammatory enzymes (Al-Khayri et al., 2022).

This study aims to evaluate the *in vivo* anti-inflammatory activity of four *Heracleum* taxa—*H. paphlagonicum* Czeczott, *H. sphondylium* subsp*. ternatum* (Velen.) Brummitt, *H. sphondylium* subsp. *elegans* (Crantz) Schübl. & G. Martens, and *H. sphondylium* subsp. *cyclocarpum*—using carrageenan-, PGE2-, and serotonin-induced paw edema models in mice. These taxa were selected for the present study due to their natural occurrence in Turkey and the lack of prior research on their anti-inflammatory properties. The primary objective is to investigate the anti-inflammatory activities of these plants, especially those abundant in coumarin derivatives, which are well-known for their significant anti-inflammatory effects. Furthermore, this study aims to validate the traditional use of *Heracleum* in managing inflammation and to examine the relationship between anti-inflammatory activity and phytochemical content.

## 2 Materials and methods

### 2.1 Plant materials

Plant materials were collected during the extended flowering period in summer from the locations specified in Table 1 and subsequently identified by Prof. Dr. Hayri Duman and Prof. Dr. Ahmet Duran. Voucher specimens are preserved in the Herbarium of the Ankara University Faculty of Pharmacy (AEF).

**TABLE 1:** Location of plants.

Plant	Location	Habitat	Date	Voucher specimen
*H. paphlagonicum*	Ilgaz, Çankırı, Turkey	Riverside, 1753 m	2019-June	AEF 28815
*H. sphondylium* subsp. *ternatum*	Ankara-Kırıkkale, Turkey	Riverside, 900 m	2019-June	AEF 28809
*H. sphondylium* subsp. *elegans*	Yenice, Karabük, Turkey	By streams in forest, 1000 m	2019-June	AEF 28814
*H. sphondylium* subsp. *cyclocarpum*	Murgul, Artvin, Turkey	By streams in forest, 2136 m	2019-July	AEF 28812

### 2.2 Extraction of the plants

Flowering aerial parts and roots were separated, dried at room temperature (∼25°C), and powdered. Dichloromethane (Sigma-Aldrich) was added to each plant material and macerated for 24 h at room temperature (∼25°C), followed by stirring in an ultrasonic bath for 1 hour. The extracts were then filtered and evaporated under vacuum at 40°C–45°C. The residual plant materials were dried, and methanol (Sigma-Aldrich) was added. The plant materials were macerated in methanol for 24 h and stirred in an ultrasonic bath for 1 hour. The extracts were filtered again and evaporated under vacuum at 40°C–45°C. Finally, the methanolic extracts were lyophilized to obtain crude extracts. The extraction solvents were chosen due to their broad extraction capabilities; methanol is a widely used solvent for polar compounds, including phenolics, flavonoids, and alkaloids, while dichloromethane is utilized to extract nonpolar compounds.

The quantities of plant materials and the extract yields are presented in Table 2.

**TABLE 2:** Crude extracts quantities with amounts of plant material for extraction.

Plant	Part of plant	Amount of plant material (g)	Extraction solvent	Amount of extract (g)	Extract yield (%)
*H. paphlagonicum*	R	175.42	DCM	4.78	2.72
			MeOH	11.05	6.30
	AE	254.18	DCM	5.40	2.13
			MeOH	24.16	9.51
*H. sphondylium* subsp. *ternatum*	R	120.37	DCM	6.57	5.46
			MeOH	10.45	8.68
	AE	149.69	DCM	6.57	4.39
			MeOH	8.04	5.37
*H. sphondylium* subsp. *elegans*	R	173.22	DCM	2.34	1.35
			MeOH	12.42	7.17
	AE	158.72	DCM	5.93	3.74
			MeOH	11.29	7.11
*H. sphondylium* subsp*. cylocarpum*	R	169.81	DCM	7.10	4.18
			MeOH	10.54	6.21
	AE	167.56	DCM	3.83	2.29
			MeOH	12.63	7.54

R: root, AE: aerial part, DCM: dichloromethane, MeOH: methanol.

### 2.3 Anti-inflammatory activity

#### 2.3.1 Animals

Male Swiss albino mice (20–25 g) were purchased from Kobay Animal Breeding Laboratory to use for tests. Animals were housed in the laboratory for 2 days and fed pellet food and water *ad libitum*. Six mice were used for each group. The current study was conducted according to international rules considering animal experiments and biodiversity rights (Kobay Animal Breeding Laboratory Ethical Council Project Number: 408).

#### 2.3.2 Preparation of the test materials

Extracts were suspended in 0.5% carboxymethyl cellulose (CMC) and administered orally to mice by gastric gavage at a dose of 100 mg/kg. Mice in the control group were received 0.5% CMC, and indomethacin (Nobel) (10 mg/kg) in 0.5% CMC was used as a reference drug. Sixty minutes after the administration of the test materials, inflammation was induced by carrageenan, prostaglandin E2, and serotonin separately.

#### 2.3.3 Carrageenan-induced hind paw edema

25 µL of carrageenan suspension (50 mg carrageenan (Sigma Co., No. C-1013) in 2.5 mL of saline) was injected into the subplantar tissues of the right hind paw of each animal. 25 μL saline solution was injected into the subplantar tissues of the right hind paw of the mice as a control. The swelling thickness of each paw was measured every 90 min using a micrometer calliper (Ozaki Co., Tokyo, Japan) for 6 h. Differences between thickness of the right and left paw were considered to indicate inflammation levels. The mean values of each group were compared and analyzed statistically (Toker et al., 2004; Yesilada and Kupeli, 2007).

#### 2.3.4 PGE2-induced hind paw edema

5 µL PGE2 solution (5 µg PGE2, Fluka Chemie AG, Art. 82,475, in 5 µL Tyrode’ solution) was injected into the subplantar tissues of the right hind paw of each mouse, and 5 µL Tyrode’ solution was injected into the subplantar tissues of the left hind paws. Paw edema differences between the right and left paws were measured with a micrometer caliper at 15-min intervals for 75 min. The mean values of the test and control groups were compared and analyzed statistically (Yeşilada and Küpeli, 2002; Akkol and Ercil, 2009).

#### 2.3.5 Serotonin-induced hind paw edema

5 µL of serotonin solution (0.5 µg serotonin creatinine sulfate, Merck, Art. 7768, in 5 µL Tyrode’s solution) was injected into the subplantar tissues of the right hind paw of each mouse. 5 μL Tyrode’s solution was injected into the subplantar tissues of the left hind paws as a control. Paw edema differences between the right and left paws were measured with a micrometer caliper every 6 min for 30 min. The mean values of the test and control groups were compared and analyzed statistically (Küpeli et al., 2002; Erdemoglu et al., 2003).

#### 2.3.6 Statistical analysis of data

Data from animal experiments were expressed as ± mean standard error (±SEM). Statistical differences between treatment and control groups were evaluated using ANOVA and Student-Newman-Keuls *post hoc* tests. A probability of *p* < 0.05 was considered significant (*p < 0.05; **p < 0.01; ***p < 0.001).

### 2.4 Phytochemical analyses

The coumarin and flavonoid-phenolic acid profiles of the plant extracts were examined using high-performance liquid chromatography (HPLC) (Agilent 1260 G1315 DAD) and an ACE5 C18 (250 × 4.6 mm; 5 μL) column.

#### 2.4.1 Dedection of coumarins

The contents of angelicin, bergapten, xanthotoxin, osthol, umbelliferone, imperatorin, isoimperatorin, deltoin, columbianetin, and isoepoxypteryxin in the extracts were investigated both qualitatively and quantitatively. Solutions of xanthotoxin, imperatorin, angelicin, and osthol were prepared in five concentrations (ranging from 0.025 to 0.5 mg/mL), while bergapten solutions were prepared in seven concentrations (ranging from 0.005 to 0.5 mg/mL). Each plant extract was prepared at a concentration of 10 mg/mL and then filtered using 0.45 μm membrane filters. The extracts and standard compounds were injected into the HPLC three times. Calibration curves for each compound were generated using peak areas at UV_254_ nm (UV_330_ nm for osthol) and their corresponding concentrations. The limit of detection (LOD) and quantification (LOQ) for the compounds were determined as signal-to-noise ratios of 3 and 10, respectively, and injected into HPLC six times during 3 days. The mobile phase consisted of 0.2% phosphoric acid in water (A) and methanol (B). Gradient elution of the mobile phase started with 55% A and 45% B, changing linearly to 34.5% A and 65.5% B in 5 minutes, linearly flowing to 33.5% A and 67.5% B between minutes 5 and 25, reaching 100% B by minutes 30. Between minutes 30 and 35, 100% B was maintained. The flow rate was set at 0.5 mL/min, and the column temperature was maintained at 40°C. The post time was set to 5 min. The maximum absorbance of xanthotoxin, imperatorin, and angelicin was measured at UV_254_ nm, while the maximum absorbance of osthol was at UV_330_ nm.

#### 2.4.2 Dedection of flavonoids and phenolic acids

Amentoflavone, apigenin, apigenin-7-*O*-*β*-glucoside, hyperoside, quercetin, isoquercetin, kaempferol, luteolin, luteolin-7-*O*-glucoside, vitexin-2-*O*-rhamnoside, and ferulic, gallic, caffeic, quinic, sinapic, syringic, and vanillic acid contents of the extracts were examined qualitatively. Gradient elution of 0.2% phosphoric acid in water (A), acetonitrile (B), and methanol (C) solvents started with A:B (90:10) and changed linearly to A:B (0:100) in 20 min 100% C flowed during the 20.01–25 min. The column temperature was 40°C, and the flow rate was 1 mL/min.

## 3 Results

The present study revealed the anti-inflammatory activities of dichloromethane and methanolic extracts *from the* roots and aerial parts of *H. paphlagonicum, H. sphondylium* subsp. *ternatum, H. sphondylium* subsp. *elegans*, *H. sphondylium* subsp. *cylocarpum* in carrageenan-, prostaglandin E2 (PGE2)-, and serotonin-induced hind paw edema models at a dose of 100 mg/kg dose. The results are presented in Tables 3–5.

**TABLE 3:** Effect of the test materials against carrageenan-induced hind paw edema in mice.

Material	Part of plant	Extraction solvent	Dose (mg/kg)	Swelling thickness (x10^−2^mm) ± SEM (% inhibition)			
				90 min	180 min	270 min	360 min
Control				46,2 ± 5.4	53.5 ± 5.9	60.1 ± 5.7	66.8 ± 6.4
*H. paphlagonicum*	R	DCM	100	49.8 ± 4.7	59.4 ± 5.2	64.3 ± 5.9	67.2 ± 5.1
		MeOH	100	47.5 ± 5.3	47.1 ± 5.4 (11.9)	53.3 ± 5.0 (11.3)	56.4 ± 4.8 (15.6)
	AE	DCM	100	48.9 ± 5.4	48.8 ± 5.1 (8.8)	52.1 ± 5.2 (13.3)	59.5 ± 4.9 (10.9)
		MeOH	100	51.4 ± 5.5	46.2 ± 5.3 (13.6)	51.5 ± 5.4 (14.3)	58.8 ± 4.8 (11.9)
*H. sphondylium* subsp. *ternatum*	R	DCM	100	42.1 ± 3.7 (8.8)	44.2 ± 3.9 (17.4)	48.1 ± 4.2 (19.9)	51.2 ± 3.9 (23.4)*
		MeOH	100	48.5 ± 3.6	45.5 ± 3.9 (14.9)	47.6 ± 3.7 (20.8)	49.8 ± 3.5 (25.4)*
	AE	DCM	100	55.7 ± 4.2	46.3 ± 4.6 (13.5)	52.3 ± 5.0 (12.9)	68.6 ± 4.9
		MeOH	100	48.0 ± 2.8	54.3 ± 3.2	61.4 ± 3.1	69.4 ± 3.5
*H. sphondylium* subsp. *elegans*	R	DCM	100	55.9 ± 3.1	55.6 ± 3.6	63.5 ± 3.8	70.3 ± 3.5
		MeOH	100	54.6 ± 5.0	59.1 ± 5.5	55.2 ± 5.1 (8.2)	57.0 ± 4.4 (14.7)
	AE	DCM	100	50.2 ± 3.5	54.2 ± 3.8	65.3 ± 4.0	70.3 ± 3.7
		MeOH	100	46.9 ± 3.1	57.8 ± 5.2	56.0 ± 5.3 (6.8)	60.1 ± 3.9 (10.0)
*H. sphondylium* subsp. *cyclocarpum*	R	DCM	100	52.1 ± 3.2	40.5 ± 4.2 (24.3)*	43.4 ± 4.6 (27.8)*	44.2 ± 4.3 (33.8)**
		MeOH	100	51.6 ± 5.1	41.3 ± 4.1 (22.8)	40.5 ± 3.9 (32.6)**	42.1 ± 4.2 (36.9)**
	AE	DCM	100	53.5 ± 3.4	55.9 ± 3.1	64.6 ± 3.6	71.4 ± 3.9
		MeOH	100	50.4 ± 3.9	55.2 ± 3.4	60.2 ± 3.9	68.5 ± 4.2
Indomethacin			10	40.3 ± 4.0 (12.8)	39.8 ± 3.7 (25.6)*	38.4 ± 3.5 (36.1)**	37.2 ± 3.4 (44.3)***

*p < 0.05; **p < 0.01; ***p < 0.001; R: root, AE: aerial part, DCM: dichloromethane, MeOH: methanol, SEM: standard error of the mean.

**TABLE 4:** Effect of the test materials on PGE2-induced paw edema in mice.

Material	Part of plant	Extraction solvent	Dose (mg/kg)	Swelling thickness (x 10^−2^mm)± SEM (inhibition %)					
				0 min	15 min	30 min	45 min	60 min	75 min
Control				3.7 ± 1.2	16.3 ± 1.5	23.7 ± 1.4	15.8 ± 1.2	12.6 ± 1.3	9.2 ± 1.1
*H. paphlagonicum*	R	DCM	100	3.9 ± 1.1	16.4 ± 1.2	20.9 ± 1.5 (11.8)	13.9 ± 1.4 (12.0)	10.9 ± 1.6 (13.5)	7.5 ± 1.2 (18.5)
		MeOH	100	3.7 ± 1.2	16.6 ± 1.4	19.8 ± 1.3 (16.5)	12.7 ± 1.6 (19.6)	9.7 ± 1.4 (23.0)	7.7 ± 1.5 (16.3)
	AE	DCM	100	4.0 ± 1.4	16.5 ± 1.6	25.1 ± 1.4	14.4 ± 1.9 (8.9)	10.7 ± 1.8 (15.1)	14.4 ± 1.6
		MeOH	100	3.9 ± 1.0	16.1 ± 1.1 (1.2)	21.3 ± 1.9 (10.1)	14.3 ± 1.7 (9.5)	19.8 ± 1.3	13.7 ± 1.6
*H. sphondylium* subsp. *ternatum*	R	DCM	100	3.7 ± 1.0	16.3 ± 1.3	18.8 ± 1.4 (20.7)	10.4 ± 1.0 (34.2)*	9.5 ± 0.6 (24.6)*	7.4 ± 0.8 (19.6)
		MeOH	100	3.9 ± 1.1	16.5 ± 1.0	18.1 ± 1.2 (23.7)	10.7 ± 0.7 (32.3)*	8.9 ± 0.9 (29.4)*	7.3 ± 1.1 (20.7)
	AE	DCM	100	4.1 ± 1.3	16.4 ± 1.9	24.7 ± 1.6	13.8 ± 1.9 (12.7)	9.7 ± 1.3 (23.0)	7.6 ± 1.0 (17.4)
		MeOH	100	4.1 ± 1.2	16.6 ± 1.2	25.6 ± 1.7	17.7 ± 1.6	17.9 ± 1.5	11.9 ± 1.7
*H. sphondylium* subsp. *elegans*	R	DCM	100	4.2 ± 1.6	15.1 ± 1.6 (7.4)	21.8 ± 1.6 (8.0)	18.8 ± 1.3	19.6 ± 1.8	11.3 ± 1.2
		MeOH	100	3.8 ± 1.1	15.7 ± 0.7 (3.7)	19.7 ± 0.6 (16.9)	13.2 ± 07. (16.5)	10.8 ± 0.8 (14.3)	8.9 ± 0.6 (3.3)
	AE	DCM	100	4.2 ± 1.3	16.9 ± 1.9	24.6 ± 1.5	17.2 ± 1.3	16.5 ± 1.8	14.8 ± 1.8
		MeOH	100	4.4 ± 1.1	16.5 ± 1.4	25.9 ± 1.8	17.5 ± 1.6	16.7 ± 1.4	14.7 ± 1.3
*H. sphondylium* subsp. *cyclocarpum*	R	DCM	100	3.5 ± 1.0 (5.4)	14.8 ± 1.1 (9.2)	16.4 ± 0.8 (30.8)*	10.5 ± 0.9 (33.5)*	8.7 ± 0.9 (30.9)**	7.1 ± 1.1 (22.8)
		MeOH	100	4.0 ± 0.9	13.6 ± 1.1 (16.6)	17.5 ± 0.8 (26.2)*	11.2 ± 1.0 (29.1)*	8.1 ± 0.8 (35.7)**	6.7 ± 0.7 (27.2)*
	AE	DCM	100	4.0 ± 1.1	16.4 ± 1.9	26.5 ± 1.4	18.6 ± 1.9	18.5 ± 1.7	13.5 ± 1.8
		MeOH	100	4.1 ± 1.1	16.5 ± 1.3	26.3 ± 1.7	18.2 ± 1.4	18.9 ± 1.9	153.±1.3
Indomethacin			10	36 ± 0.5 (2.7)	13.9 ± 1.1 (14.7)	15.2 ± 1.0 (35.9)**	9.9 ± 0.9 (37.3)**	7.4 ± 0.7 (41.3)***	6.1 ± 0.8 (33.7)**

*p < 0.05; **p < 0.01; ***p < 0.001; R: root, AE: aerial part, DCM: dichloromethane, MeOH: methanol, SEM: standard error of the mean.

**TABLE 5:** Effect of the test materials on serotonin-induced paw edema in mice.

Materials	Part of plant	Extraction solvent	Dose (mg/kg)	Swelling thickness (x 10^−2^mm)± S.E.M. (inhibition%)					
				0 min	6 min	12 min	18 min	24 min	30 min
Control				4.2 ± 0.9	9.8 ± 1.1	15.7 ± 1.4	20.2 ± 1.3	22.9 ± 1.1	25.5 ± 1.3
*H. paphlagonicum*	R	DCM	100	4.3 ± 0.5	10.1 ± 0.7	12.9 ± 1.2 (17.8)	18.1 ± 1.1 (10.4)	20.4 ± 1.3 (10.9)	25.8 ± 1.2
		MeOH	100	4.9 ± 0.9	10.8 ± 1.1	18.8 ± 1.4	25.7 ± 1.6	27.4 ± 1.3	29.8 ± 1.5
	AE	DCM	100	4.6 ± 0.8	12.3 ± 1.2	15.8 ± 1.1	22.8 ± 1.2	25.8 ± 1.8	28.5 ± 1.7
		MeOH	100	4.8 ± 1.1	11.6 ± 1.3	17.1 ± 1.1	18.9 ± 1.5 (6.4)	20.6 ± 1.0 (10.0)	27.0 ± 1.2
*H. sphondylium* subsp. *ternatum*	R	DCM	100	4.7 ± 0.7	10.1 ± 1.4	14.2 ± 1.5 (9.6)	18.3 ± 1.2 (9.4)	19.2 ± 1.6 (16.2)	26.0 ± 1.1
		MeOH	100	4.5 ± 0.7	9.2 ± 1.1 (6.1)	13.4 ± 1.2 (14.6)	16.6 ± 1.4 (17.8)	18.9 ± 1.1 (17.5)	20.8 ± 1.0 (18.4)
	AE	DCM	100	4.9 ± 0.8	9.9 ± 1.7	14.9 ± 1.5 (5.1)	19.5 ± 1.4 (3.5)	19.9 ± 1.9 (13.1)	28.3 ± 1.7
		MeOH	100	5.3 ± 1.4	11.0 ± 1.3	17.7 ± 1.4	22.9 ± 1.5	26.1 ± 1.7	29.3 ± 1.5
*H. sphondylium* subsp. *elegans*	R	DCM	100	4.6 ± 0.5	10.8 ± 0.9	16.1 ± 1.3	21.7 ± 1.2	23.2 ± 1.3	26.9 ± 1.2
		MeOH	100	5.4 ± 1.3	12.1 ± 1.6	18.3 ± 1.2	23.4 ± 1.4	25.2 ± 1.3	27.9 ± 1.1
	AE	DCM	100	5.0 ± 1.1	9.5 ± 1.3 (3.1)	13.6 ± 1.2 (13.4)	18.8 ± 1.1 (6.9)	24.2 ± 1.5	25.6 ± 1.5
		MeOH	100	4.3 ± 0.6	11.5 ± 0.9	16.9 ± 1.2	21.5 ± 1.4	23.1 ± 1.5	26.8 ± 1.4
*H. sphondylium* subsp. *cyclocarpum*	R	DCM	100	4.4 ± 0.7	9.9 ± 0.8	13.3 ± 1.1 (15.3)	17.5 ± 1.2 (13.4)	20.0 ± 1.5 (12.7)	22.9 ± 1.1 (10.2)
		MeOH	100	5.1 ± 1.2	10.6 ± 1.4	14.8 ± 1.3 (5.7)	19.4 ± 1.1 (3.9)	18.8 ± 1.5 (17.9)	22.5 ± 1.3 (11.8)
	AE	DCM	100	4.1 ± 0.7	10.9 ± 1.1	17.9 ± 1.6	27.4 ± 1.8	25.2 ± 1.4	27.6 ± 1.4
		MeOH	100	4.1 ± 0.5	13.2 ± 0.9	16.3 ± 1.3	21.3 ± 1.2	28.9 ± 1.3	28.3 ± 1.5
Indomethacin			10	3.9 ± 0.4 (7.1)	7.2 ± 0.6 (26.5)*	10.9 ± 1.1 (30.6)**	15.4 ± 0.9 (23.8)*	16.2 ± 0.7 (29.3)**	18.8 ± 0.4 (26.2)**

*p < 0.05; **p < 0.01; ***p < 0.001; R: root, AE: aerial part, DCM: dichloromethane, MeOH: methanol, SEM: standard error of the mean.

The evaluation of the carrageenan-induced hind paw edema test yielded the most significant results among the three activities assessed, demonstrating pronounced effects for both indomethacin as the positive control and the extracts. The methanolic root extract of *H. sphondylium* subsp. *cyclocarpum* yielded exceptional results, with inhibition values between 22.8% and 36.9% on carrageenan-induced inflammation. Additionally, the dichloromethane extract from the same subspecies demonstrated an anti-inflammatory effect, with inhibition rates ranging from 24.3% to 33.8%. The root extract from *H. sphondylium* subsp. *ternatum* also displayed notable activity and was recorded as the second-highest anti-inflammatory effect in the study. The dichloromethane and methanol extracts of the plant exhibited inhibition rates ranging from 17.4% to 23.4% and 14.9%–25.9%, respectively, demonstrating a significant level of efficacy. In the same experiment, indomethacin inhibited 12.8%–44.3% of carrageenan-induced inflammation (Table 3). Conversely, *H. sphondylium* subsp. *elegans* root and aerial part extracts (utilizing dichloromethane and methanol) did not show statistically significant results. Their effects were not comparable to those observed in the other tested species or the established positive control (see Table 3 for detailed data). In addition, *H. paphlagonicum.* displayed anti-inflammatory activity starting at the 180th minute after administration, with an 11.9% inhibition that persisted until the 270th minute, then increased to 15.6% by the 360th minute, which may not be particularly significant, highlighting variability in responses among different taxa.

*H. sphondylium* subsp. *cyclocarpum* noteworthy effect persisted in the prostaglandin-induced inflammation test. Same as in the carrageenan-induced paw edema test *H. sphondylium* subsp. *cyclocarpum* root dichloromethane and methanol extracts demonstrated substantial inhibitory activities when compared to the other taxa, with 5.4%–33.5% and 16.6%–35.7% inhibition rates, respectively. The dichloromethane extract is considered the most potent active extract, clearly exhibiting exceptional inhibitory activity against inflammation. This activity commenced primarily at the 30th minute, achieving a 30.8% inhibition, and remained relatively stable until the 60th minute. By the 75th minute, there was a slight decrease in inhibition to 22.8%. In the methanol extract, the observed effects were similar to those of the dichloromethane extract, with a few minor differences. Notably, the inhibition rate at the 30th minute was slightly lower (26.2%) than that of the dichloromethane extract. However, slightly higher inhibition rates (35.7%; 27.2%) were recorded at the 60th and at the 75th minutes. While the root was found to be the most potent active extract, no significant differences were observed in the aerial parts of the *H. sphondylium* subsp. *cyclocarpum* extracts. Roots of *H. sphondylium* subsp. *ternatum* also displayed similar anti-inflammatory activity to those of *H. sphondylium* subsp. *cyclocarpum* in the PGE2-induced inflammation test model; however, the inhibition percentages were lower. The highest levels of inflammation inhibition were observed at the 60th minute for both the dichloromethane and methanol extracts, with inhibition percentages of 34.2% and 32.3%, respectively. The evaluation of the extracts obtained from the aerial parts clearly indicates that the methanol extract demonstrates a measurable effect. This effect initiates at 45th minutes, achieves a peak inhibition of 20% at 60 min, and decreases notably by the 75th minute. The methanolic extract of the roots of *H. sphondylium* subsp. *elegans* demonstrated minimal inhibitory activity that lacked statistical significance. Additionally, the remaining extracts obtained from both the aerial parts and the roots of the subspecies showed no detectable activity in the PGE2-induced inflammatory test model. However, weak activity was observed when treated with *H. paphlagonicum*; the methanolic extract demonstrated inhibition values ranging from 16.3% to 23.0%, while the dichloromethane extract showed inhibition values between 11.8% and 18.5% (Table 4).

However, in the model involving serotonin-induced hind paw edema, the reference drug indomethacin produced an inhibition range of 7.1%–30.6%. It's noteworthy that none of the extracts from the studied plants was able to exhibit any inhibitory activity against edema in this specific model (as shown in Table 5). This lack of effectiveness suggests that different inflammatory pathways may respond distinctly to the extracts, underscoring the complexity of their pharmacological profiles and the need for further research to elucidate these mechanisms.

**FIGURE 1:**
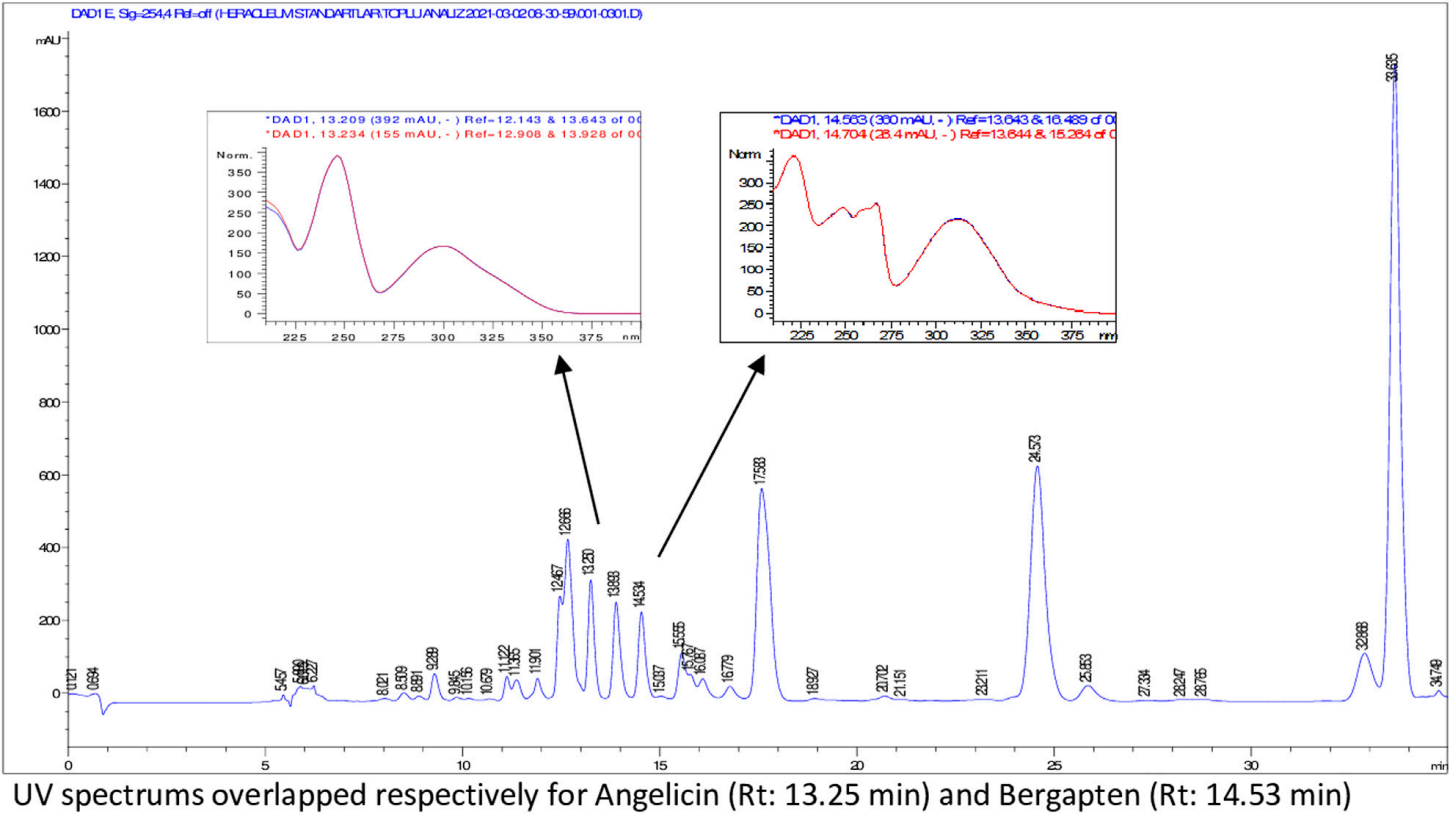
HPLC chromatogram of dichloromethane extract of *H. paphlagonicum* roots (UV_254_).

**FIGURE 2:**
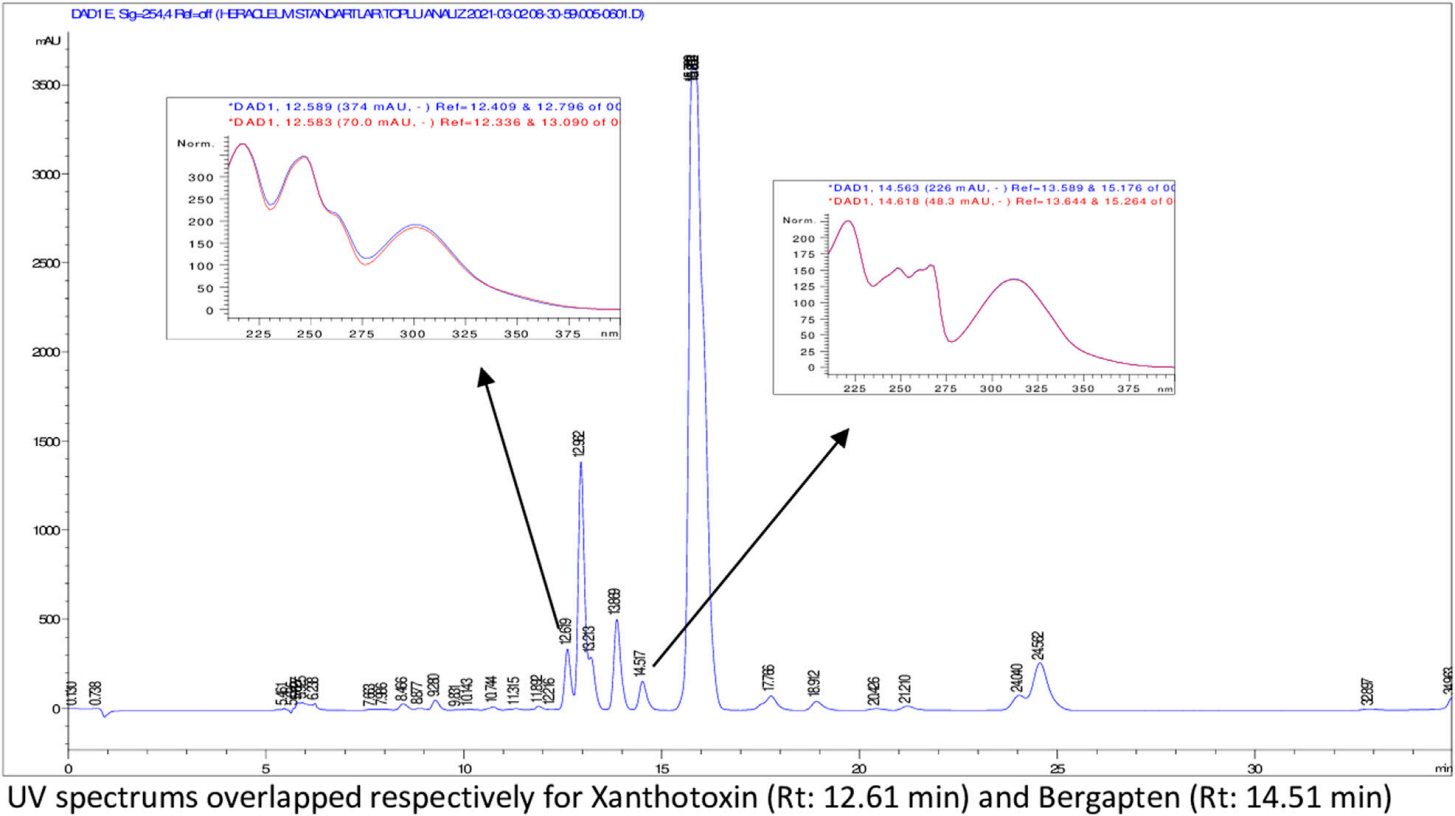
HPLC chromatogram of dichloromethane extract of *H. sphondylium* subsp. *ternatum* roots (UV_254_).

**FIGURE 3:**
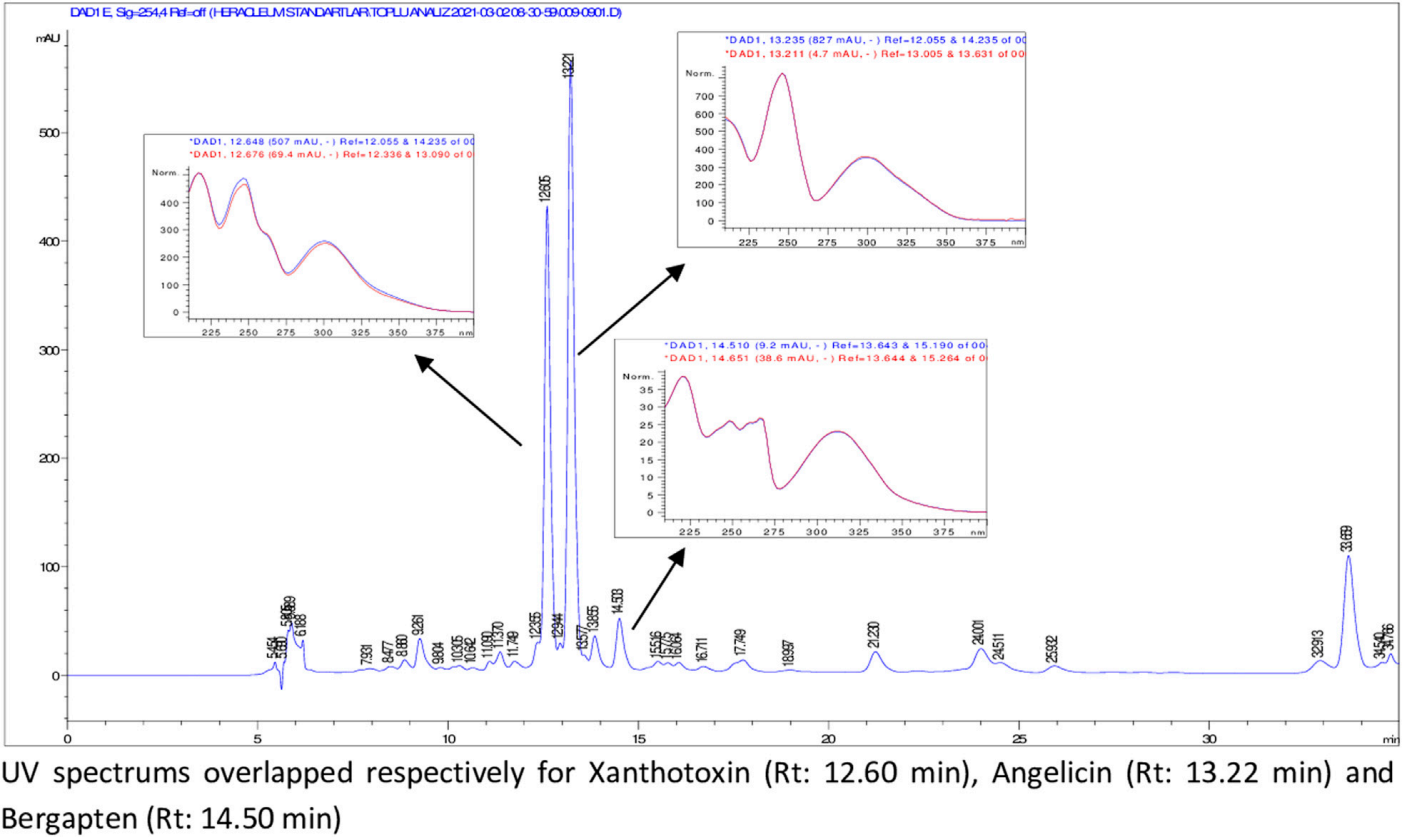
HPLC chromatogram of dichloromethane extract of *H. sphondylium* subsp. *elegans* roots (UV_254_).

**FIGURE 4:**
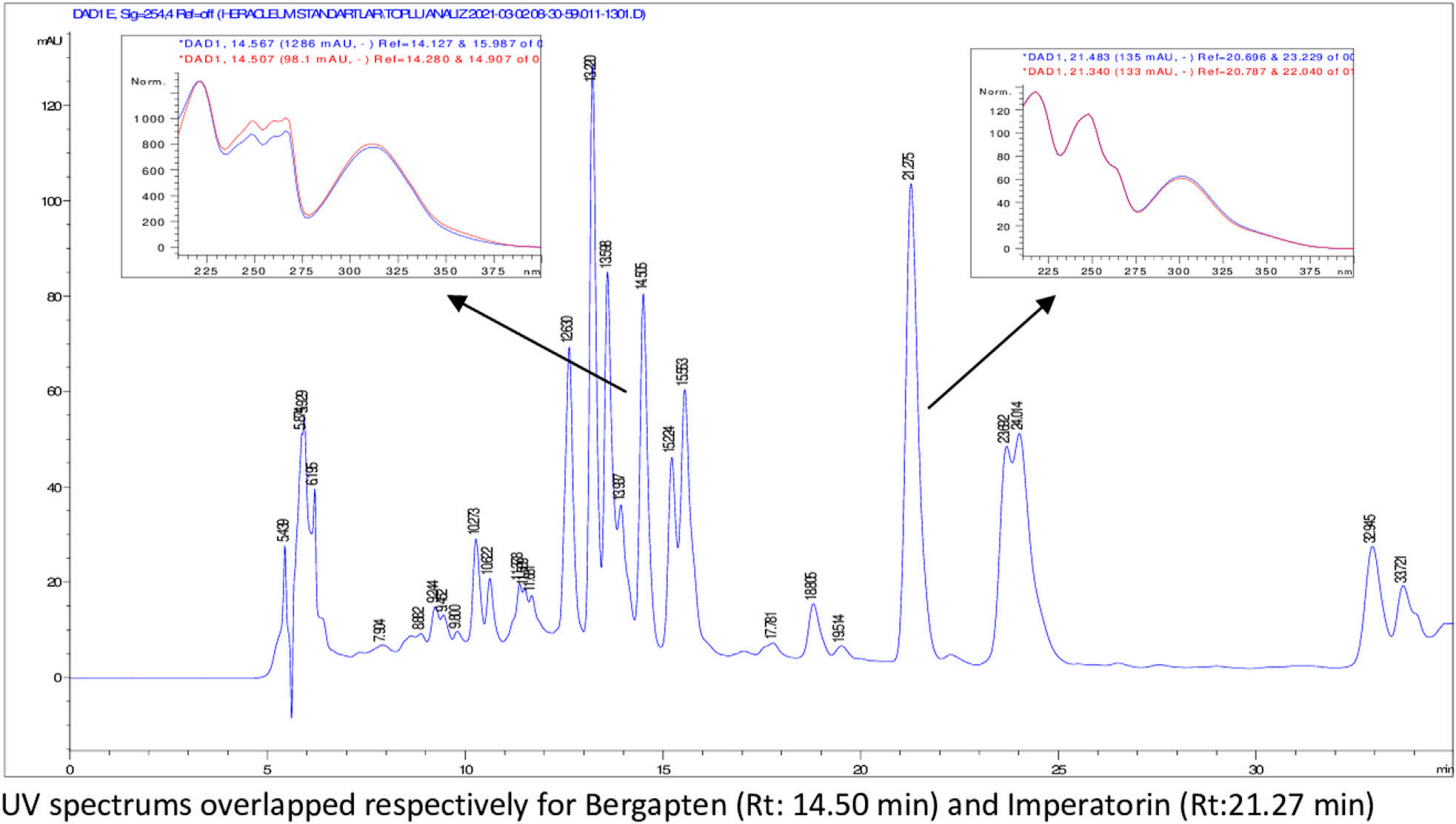
HPLC chromatogram of dichloromethane extract of *H. sphondylium* subsp. *elegans* aerial parts (UV_254_).

**FIGURE 5:**
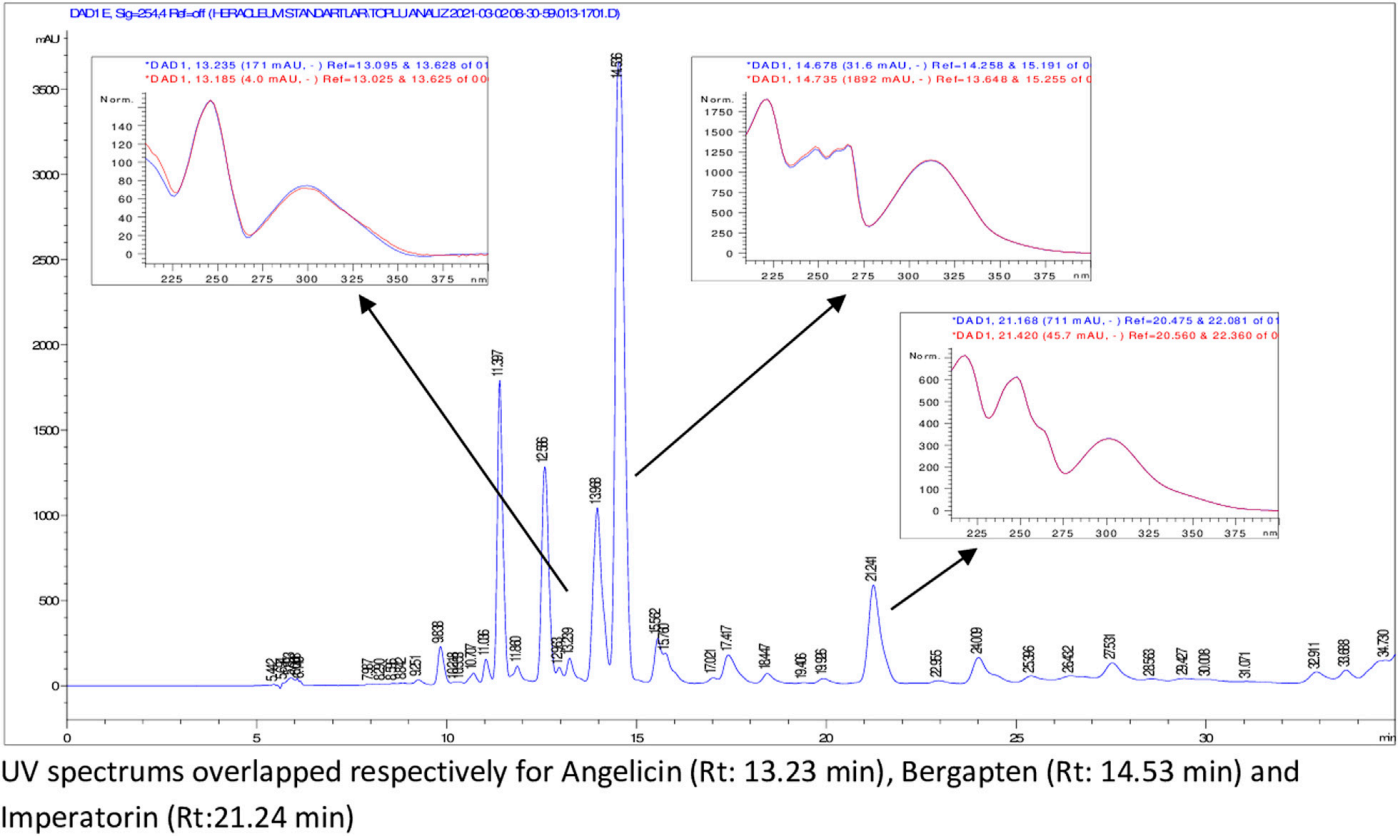
HPLC chromatogram of dichloromethane extract of *H. sphondylium* subsp. *cyclocarpum* roots (UV_254_).

**FIGURE 6:**
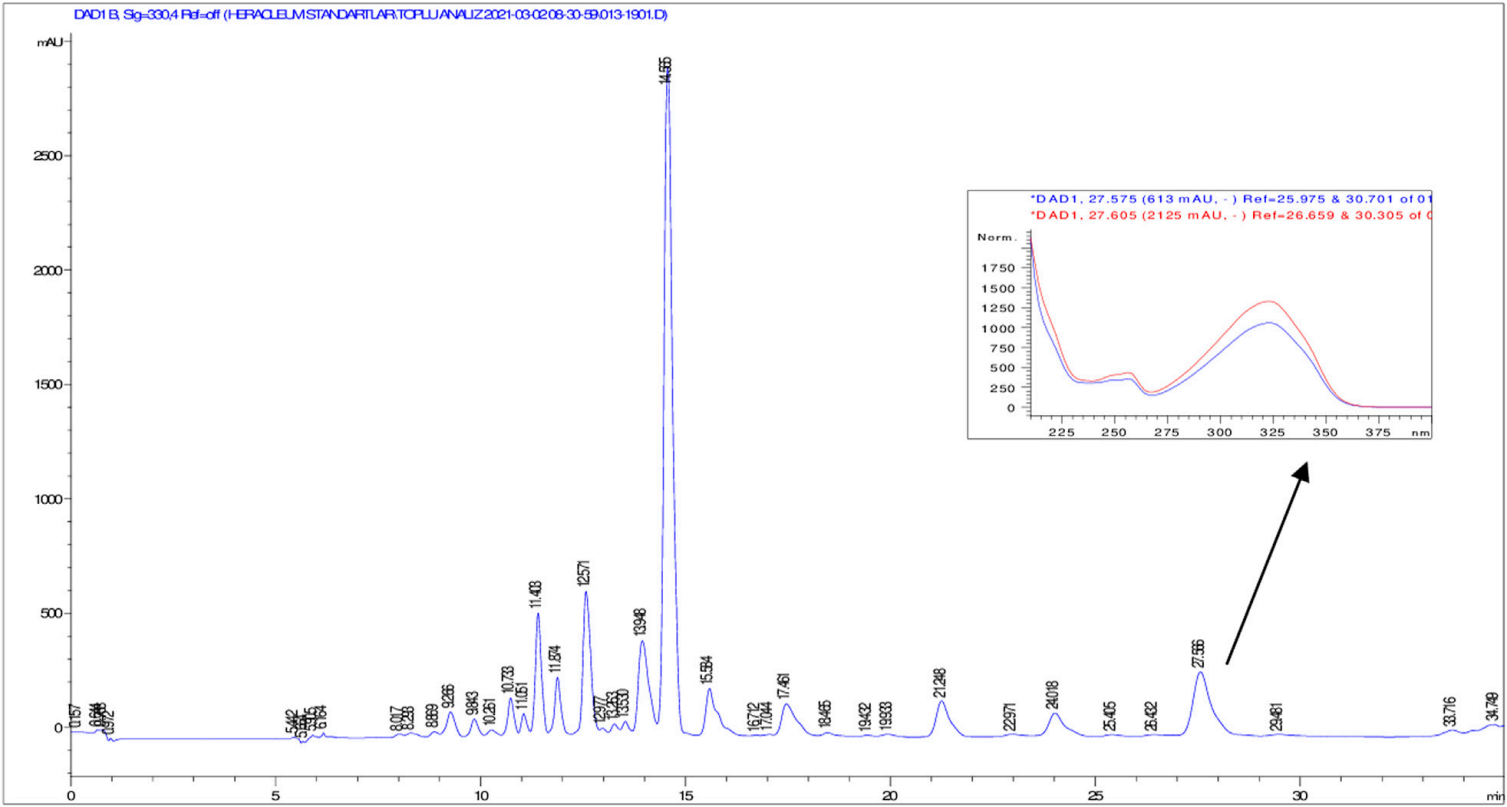
HPLC chromatogram of dichloromethane extract of *H. sphondylium* subsp. *cyclocarpum* roots (UV_330_).

According to HPLC analyses, flavonoids, phenolic acids, and several coumarins including umbelliferone, isoimperatorin, deltoin, columbianetin, and isoepoxypteryxin, were not detected in the plant extracts. HPLC chromatograms of the extracts with detected compounds are presented in Figures 1–6.

*H. sphondylium* subsp. *cyclocarpum* roots exhibited the highest bergapten content (0.49% mg/g), while osthole, a 7-methoxy coumarin derivative, was deteced in only the dichloromethane extract of *H. sphondylium* subsp. *cyclocarpum* roots (0.05% mg/g). Xanthotoxin was identified in *H. sphondylium* subsp. *elegans* and *H. sphondylium* subsp. *ternatum* roots (0.06% and 0.04% mg/g, respectively). Angelicin quantities of *H. paphlagonicum*, *H. sphondylium* subsp. *elegans*, and *H. sphondylium* subsp. *cyclocarpum* roots were found to be comparable (0.04%, 0.04%, and 0.02% mg/g, respectively). Imperatorin was detected in *H. sphondylium* subsp. *elegans* aerial parts and *H. sphondylium* subsp. *cyclocarpum* roots (0.02% and 0.14% mg/g, respectively). The coumarin quantities of the extracts, along with the LOD and LOQ values of the compounds, are given in Table 6.

**TABLE 6:** Quantities of detected compounds in plant materials.

Material	Part of plant	Quantities of the compounds in plant materials (% mg/g)				
		Xanthotoxin	Angelicin	Bergapten	Imperatorin	Osthole
*H.paphlagonicum*	R	-	0.0399 ± 0.0001	0.0125 ± 0.0001	-	-
*H.sphondylium* subsp.*ternatum*	R	0.0361 ± 0.0001	-	0.0089 ± 0.0000	-	-
*H.sphondylium* subsp.*elegans*	R	0.0586 ± 0.0001	0.0380 ± 0.0006	0.0010 ± 0.0000	-	-
	AE	-	-	0.0047 ± 0.0000	0.0238 ± 0.0000	-
*H.sphondylium* subsp.*cyclocarpum*	R	-	0.0218 ± 0.0001	0.4982 ± 0.0015	0.1411 ± 0.0093	0.0522 ± 0.0006
LOD		0.00079	0.00079	0.00044	0.00022	0.00559
LOQ		0.0024	0.0024	0.00147	0.00075	0.01863

R: root, AE: aerial part, LOD: limit of detection, LOQ: limit of quantification.

## 4 Discussion

In light of our results, the roots of *H. sphondylium* subsp. *cyclocarpum* exhibited significant anti-inflammatory activity by inhibiting carrageenan-, PGE2-, and serotonin-induced edema. Additionally, roots of *H*. *sphondylium* subsp. *ternatum* demonstrated notable activity against carrageenan- and PGE2-induced inflammation. Although no previous studies have specifically addressed the anti-inflammatory effects of the investigated taxa, several *in vitro* and *in vivo* studies have reported the anti-inflammatory properties of various *Heracleum* species.

The essential oil and hydroalcoholic extract of *H. persicum* fruit were evaluated for their anti-inflammatory activity. The essential oil significantly inhibited carrageenan-induced paw edema in rats at doses of 100 and 200 mg/kg, while the hydroalcoholic extract showed inhibition at a dose of 400 mg/kg (Hajhashemi et al., 2009). Additionally, the aqueous-alcoholic extract of the leaves and stems of *H. persicum* was found to reduce serum levels of interleukin (IL)-6, IL-1β, and tumor necrosis factor (TNF)-α in rats with gentamicin-induced nephrotoxicity (Akbaribazm et al., 2021).

Kim et al. (2019) investigated the anti-inflammatory effects of an aqueous alcoholic extract of *H. moellendorffii* roots, reporting significant inhibition of pro-inflammatory mediators and cytokines through the suppression of nuclear factor-κB (NF-κB) and mitogen-activated protein kinase (MAPK) pathways, alongside the activation of ROS/Nrf2/HO-1 signaling. Furthermore, Hong et al. (2024) demonstrated that this extract reduced TNF-α and IL-6 levels in a dose-dependent manner in mice with induced neuroinflammation. Jang and Lee (2023) also found that the methanolic extract of the aerial parts of *H. moellendorffii* suppressed pro-inflammatory cytokine production by reducing inflammatory gene expression in lipopolysaccharide-induced RAW264.7 murine macrophage cells.

The ethanolic extract of the aerial parts of *H. dissectum* (50 mg kg^-1^/day) significantly reduced serum levels of IL-1β and myeloperoxidase, along with the mRNA expression of inflammatory response genes in epididymal white adipose tissue in high-fat diet-induced obese mice (Son et al., 2021).

The aqueous-alcoholic extract of *H. vicinium* Boiss leaves has been shown to reduce the expression of inflammation-related genes, such as IL-6 and TNF-α, in zebrafish (Liu et al., 2023).

The anti-inflammatory activities of the methanol extracts from the leaves, roots, and seeds of *H. candolleanum* were evaluated through protein denaturation, stabilization of human red blood cell (HRBC) membranes, and antiproteinase activity. At a concentration of 500 μg/mL, the extracts inhibited bovine serum albumin denaturation by 67%, 59%, and 43% for the root, seed, and leaf extracts, respectively. Similarly, the stabilization of HRBC membranes at the same concentration was 71%, 63%, and 48%, respectively. Regarding antiproteinase activity, the root extract exhibited the highest inhibition rate at 65%, followed by the seed and leaf extracts with inhibition rates of 57% and 41% (Aravind et al., 2024).

The 10% and 40% aqueous extracts prepared from the aerial parts of H. *lasiopetalum* Boiss improved ulcer healing by reducing inflammation in rats with acetic acid-induced ulcerative colitis (Malekshahi et al., 2024).

In our study, the phenolic compounds and coumarins in the extracts were also analyzed. While none of the phenolic compounds have been detected in the extracts, the roots of *H. sphondylium* subsp. *cyclocarpum*, which exhibited the highest anti-inflammatory activity, contained higher levels of coumarins, particularly bergapten and imperatorin, than other extracts.

Coumarins are a group of natural compounds that have garnered attention for their various health benefits, particularly their anti-inflammatory properties. Coumarins are distinguished by their benzopyrone ring structure. These compounds have attracted considerable scientific interest due to their remarkable anti-inflammatory properties and additional biological activities (Bansal et al., 2013). Many studies have explored the mechanisms through which coumarins exert their anti-inflammatory effects, revealing that they interact with several critical molecular targets, involve several molecular pathways and inhibit pro-inflammatory mediators. Specifically, coumarins inhibit the activity of lipoxygenase (LOX) and cyclooxygenase (COX), two critical enzymes involved in inflammation, as well as inducible nitric oxide synthase (iNOS). Inhibiting the essential enzymes COX and LOX is crucial for synthesising inflammatory mediators like prostaglandins and leukotrienes. This mechanism is one of the most reported ways for coumarin derivatives to alleviate inflammation, resulting in reduced inflammatory response and decreased swelling and inflammation (Apweiler et al., 2022; Ghosh et al., 2023). Furthermore, these compounds may lower the production of reactive oxygen species (ROS), which are the main contributors to inflammation and oxidative stress (Mishra et al., 2024). They mitigate the formation of superoxide anions generated by activated neutrophils. Beyond these mechanisms, coumarins are recognized for their powerful radical scavenging capabilities and their ability to inhibit lipid peroxidation, a process that can lead to cellular damage (Bansal et al., 2013; Grover and Jachak, 2015). They can also directly engage with cellular receptors to influence signalling cascades associated with inflammation (Kirsch et al., 2016). This interaction disrupts the production of pro-inflammatory cytokines and enzymes, ultimately leading to a reduction in inflammation. This intricate interplay of biochemical interactions positions coumarins as promising candidates for further exploration in anti-inflammatory therapeutics. All these mechanisms highlight the potential of coumarins in managing various inflammatory conditions (Hadjipavlou-Litina et al., 2007; Jarić et al., 2015; Grover and Jachak, 2015). Research utilizing several *in vitro* and *in vivo* models has also revealed that coumarins exhibit anti-inflammatory mechanisms through multiple pathways. These pathways include Toll-like receptors (TLR), the Janus Kinase/Signal Transducer and Activator of Transcription (JAK/STAT) pathway, inflammasomes, mitogen-activated protein kinase (MAPK), nuclear factor kappa-light-chain-enhancer of activated B cells (NF-κB), and the transforming growth factor beta/small mothers against decapentaplegic (TGF-β/SMAD) pathway (Rostom et al., 2022) as well as Nrf2 signaling pathway (Di Stasi, 2023).

Bergapten and imperatorin identified in the roots of *H. sphondylium* subsp. *cyclocarpum*, along with other coumarin compounds isolated from various *Heracleum* species, have been shown to exhibit anti-inflammatory activities in previous studies.

Dehydrogeijerin isolated from the leaves of *H. mollendorffii,* reduced the production of cyclooxygenase (COX)-2, pro-inflammatory cytokines, nitric oxide (NO), and inducible nitric oxide synthase (iNOS) in RAW264.7 cells (Bae et al., 2012).

An anti-inflammatory alkaloid, dissectumide A, and furanocoumarins—specifically, 3-methoxy-4-β-glucopyranosyloxy propiophenone and apterin—isolated from the roots of *H. sphondylium* subsp. *elegans*, along with sphondin obtained from *H. sphondylium*, displayed strong inhibitory activity on NO production in RAW264.7 cells (Yang et al., 2002; Zhang et al., 2017). Additionally, sphondin has inhibited IL-1β-induced COX-2 and PGE-2 expression in A549 cell lines, as well as LPS-induced nitric oxide synthase release in RAW 264.7 cells, and suppressed coumarin-7-hydroxylase activity in mouse microsomes (Yang et al., 2002).

Studies suggest that bergapten exhibits anti-inflammatory activity through the inhibition of inflammatory cytokines and mediators. Bergapten, isolated from *H. nepalense* D. Don roots, inhibited the production of lipopolysaccharide-induced pro-inflammatory cytokines, including TNF-α and IL-6, in human peripheral blood mononuclear cells (Bose et al., 2011). Aidoo et al. (2021) further evaluated the anti-inflammatory effect of bergapten, demonstrating membrane-stabilizing effects with IC_50_ values showing stronger inhibitory activity than diclofenac sodium in hypotonicity- and heat-induced hemolysis models. Bergapten also reduced protein denaturation in a concentration-dependent manner. Bergapten (10 mg/kg) decreased levels of malondialdehyde, IL-6, IL-1β, TNF-α, NF-κB, and tumor growth factor (TGF)-β1 in rats with cyclophosphamide-induced kidney inflammation (Mohsin et al., 2024). In a temporomandibular joint osteoarthritis rat model, bergapten displayed chondroprotective effects by reducing pro-inflammatory mediators and increasing type II collagen, bone volume, and trabecular number of condyles (Shen et al., 2023). Additionally, bergapten inhibited COX-2 activity and suppressed the NF-κB and MAPK signaling pathways in LPS-induced acute depression mouse models, indicating its broad anti-inflammatory potential (Yan et al., 2023).

Imperatorin displays an anti-inflammatory mechanism that targets various pro-inflammatory mediators and signaling pathways involved in the inflammatory response, similarly to the action of bergapten. Imperatorin suppressed the expression of COX-2 and iNOS in LPS-stimulated RAW 264.7 cells, even at low concentrations (Lamichhane et al., 2023). Jiang et al. (2023) reported that treatment with imperatorin inhibited the expression of IL-12p40, IL-6, TNF-α, and IL-1β in LPS-induced bone marrow-derived macrophages. Kundu et al. (2024) found that administration of imperatorin (10 mg/kg) alleviated renal inflammation in diabetic mice with kidney injury by reducing the levels of TNF-α, IL-1β, and phosphorylated NF-κB (p65) protein.

Osthole and angelicin, which are additional coumarin derivatives identified in *H. sphondylium* subsp. *cyclocarpum*, demonstrate anti-inflammatory properties. Although research on angelicin is limited, the study indicated that it exhibited an anti-inflammatory effect in LPS-induced macrophages and in cases of LPS-induced acute lung injury. Notably, angelicin effectively inhibited the NF-κB, p38, and JNK pathways while leaving the ERK pathway unaffected (Liu et al., 2013). Several studies demonstrate the anti-inflammatory activity of osthole. One study reported that osthole’s anti-inflammatory effects involve reducing inflammatory proteins and inhibiting MAPK and NLRP3 activation. MAPK pathways are vital for inflammation and some coumarins can inhibit MAPK, JAK/STAT, and NF-κB pathways. NF-κB which is vital for inflammatory responses, regulates genes involved in immunity and inflammation, and its dysregulation is linked to inflammatory disorders, making it a target for anti-inflammatory therapies (Rahman and Fazal, 2011). Osthole has been shown to inhibit NF-κB signaling and reduce cytokine production in various *in vitro* models, including hepatocytes and macrophages (Huang et al., 2017; Zhao et al., 2019). Tao et al. (2019) found that osthole significantly reverses pro-inflammatory cytokine production and oxidative stress by inhibiting NF-κB signaling and upregulating Nrf2. Additionally, docking studies indicate that osthole binds to the p65 subunit of NF-κB, preventing its DNA binding. Osthole demonstrates reducing in inflammation through NF-κB inhibition. Furthermore osthole reduce TNF-α and IL-6 release from adipocytes, correlating with lower NF-κB/p65 levels and increased PPAR-α/γ expression (Zhang et al., 2015; Rostom et al., 2022; Saadati et al., 2024).

In conclusion, the study overall emphasized significant disparities in anti-inflammatory potency among the four *Heracleum* taxa investigated—three subspecies of *H. sphondylium* and *H. paphlagonicum*. The results unequivocally identified *H. sphondylium* subsp. *cyclocarpum* as the most effective taxon, while *H. sphondylium* subsp. *ternatum* was acknowledged as the second most effective. In contrast, *H. sphondylium* subsp. *elegans* did not demonstrate any significant anti-inflammatory effect.The findings support the traditional use of *Heracleum* species in various inflammatory diseases. Considering the anti-inflammatory activities of plants within the *Heracleum* genus alongside the coumarin derivatives, it is noteworthy that the underlying pathways of their mechanisms of action are comparable. This suggests that the observed anti-inflammatory activity in *Heracleum* species can largely be attributed to coumarins. The significant anti-inflammatory activity observed in the roots of *H. sphondylium* subsp. *cyclocarpum* emphasizes the importance of further investigating both the mechanisms of action and the phytochemical profile of this plant. The investigation of the effects of the plant on various inflammatory diseases, along with a detailed examination of the mechanisms of action, could lead to promising results from a pharmacological perspective. Future investigations are planned to be employed for isolation and identification of the specific compound(s) responsible for the notable anti-inflammatory effect of *H. sphondylium* subsp. *cyclocarpum*, and, to elucidate the underlying mechanisms of action through *in vitro* experimental protocols. These studies are considered to focus on analyzing enzyme activity and may include *ex vivo* experiments to comprehensively understand the therapeutic potential of this subspecies and its phytochemical constituents.

## Data Availability

The original contributions presented in the study are included in the article/supplementary material, further inquiries can be directed to the corresponding authors.
